# Perineural Invasion in Adenoid Cystic Carcinoma of the Salivary Glands: Where We Are and Where We Need to Go

**DOI:** 10.3389/fonc.2020.01493

**Published:** 2020-08-18

**Authors:** Xiaohao Liu, Xiaojun Yang, Chaoning Zhan, Yan Zhang, Jin Hou, Xuemin Yin

**Affiliations:** Department of Oral and Maxillofacial Surgery, Nanfang Hospital, Southern Medical University, Guangzhou, China

**Keywords:** adenoid cystic carcinoma, salivary glands, perineural invasion, tumor microenvironment, perineurium barrier

## Abstract

Adenoid cystic carcinoma of the salivary gland (SACC) is a rare malignant tumors of the head and neck region, but it is one of the most common malignant tumors that are prone to perineural invasion (PNI) of the head and neck. The prognosis of patients with SACC is strongly associated with the presence of perineural spread (PNS). Although many contributing factors have been reported, the mechanisms underlying the preferential destruction of the blood-nerve barrier (BNB) by tumors and the infiltration of the tumor microenvironment by nerve fibers in SACC, have received little research attention. This review summarizes the current knowledge concerning the characteristics of SACC in relation to the PNI, and then highlights the interplay between components of the tumor microenvironment and perineural niche, as well as their contributions to the PNI. Finally, we provide new insights into the possible mechanisms underlying the pathogenesis of PNI, with particular emphasis on the role of extracellular vesicles that may serve as an attractive entry point in future studies.

## Introduction

There are three main ways by which tumors spread: direct invasion into adjacent tissues, hematogenous metastasis, and lymphatic spread; however, perineural invasion (PNI) is considered a fourth route of dissemination, which can be of great significance in the invasion and metastasis of tumors. PNI has been emerging as a significant pathologic feature of many malignant tumors, including those of the pancreas, prostate, colon, rectum, and head and neck, etc. (1). There is a high incidence of PNI in many of these malignancies, and this feature has become an indicator of poor prognosis and is associated with reduced survival (2).

SACC is one of the most common malignant salivary gland tumors, comprising 10% of all salivary gland neoplasms, which is characterized by PNI, strong invasiveness, and hematogenous metastasis (3). With respect to the primary site of tumor, there are various anatomical locations although most of them arise from the small salivary glands (75.4%). In the major salivary glands, 53.3% of tumors involve the submandibular gland, while 46.7% involve the parotid gland (4). Moreover, SACC comprises 4% of all salivary gland tumors, as well as 7.5% of all epithelial salivary gland malignancies (5). SACC generally has an indolent clinical course; nevertheless, advanced tumors may cause pain and/or nerve paralysis, as SACC has a tendency to directly invade the adjacent nerve sheaths close to the primary tumor and spreads along the nerve, a condition referred to as PNI and perineural spread (PNS), respectively.

The 5-year survival rate is relatively high in SACC patients, in contrast with the poor prognosis associated with other epithelial malignancies (5); however, the recurrence rate is also high in SACC, and closely related to PNI (6). Earlier studies suggested that lymph node involvement is uncommon in SACC, but Amit et al. found that the incidence of occult neck metastases among patients with ACC is 17% by retrospective multicentered study (7). Distant metastases are most frequently detected in the lungs, followed by bones, liver, skin, and breasts. The diagnostic transfer rate of SACC ranges from 25 to 55% (8). Distant metastases from SACC primary tumors can remain asymptomatic for long periods of time (6). Even when the tumor have been completely removed, with no recurrence at the primary tumor site, SACC still has a high tendency to spread to the other body parts over a period of time, which is the major cause of death in patients with SACC. However, outcome in SACC is significantly associated with the involvement of margins. Amit et al. analyzed the data of 507 patients with adenoid cystic carcinoma, and suggested that the positive edge is related to the worst outcome, while the negative edge and the near edge are related to the improved result, but not the distance of the tumor (9).

PNI is significantly correlated with both distant metastasis and unfavorable disease outcomes (10). Notably, in one series of patients with clear surgical margins, 80% of patients harboring PNI eventually progressed to local or distant recurrence, compared with only 27% of patients without PNI (11). Given that microscopic invasion of the cancer cells into the surrounding nerve tissues is assumed to occur via the “path of least resistance” it is often a challenge for a surgeon to detect and determine the resection border during surgery. Due to its microscopic size, CAT (computerized axial tomography), and PET (positron emission tomography) scans may also fail to detect PNI. However, Singh et al. have described that MRI can observe the enlargement and enhancement of the image of nerves that have been invaded by the tumor. This can assess whether the tumor has nerve invasion to a certain extent due to its high soft tissue contrast. Compared with CT, MRI is more sensitive to detect perineural proliferation but CT is complementary to MRI and can be used to assess local skeletal changes (12). There have been several studies concerning the statistical association between margin status and PNI. One such study demonstrated that PNI was the strongest prognostic indicator in their series of patients with SACC; however, no specific *p*-value was presented to support their claims (8). In another study, Jang et al. demonstrated that SACC with PNI subsequently progressed to metastasis, while no distant metastasis was observed in those without PNI (13). Multivariate analysis revealed that PNI was a significant predictive factor in distant metastasis (14). These results indicate that PNI potentiates distant metastatic progression, thus influencing patient outcomes.

Given that PNI is closely related to the prognosis of SACC patients, an in-depth exploration of the underlying mechanisms is needed to identify predictors of this condition, and screen out markers that predict tumor prognosis, which may contribute to the development of novel targeted drugs and reduction in recurrence risk in patients with SACC.

Currently, there are two prominent theories on the pathogenesis of PNI: one is the “path of low resistance” and the other is reciprocal signaling interactions. Due to the anatomical proximity between the salivary glands and some cranial nerves, such as the facial nerve, trigeminal nerve, hypoglossal nerve, and glossopharyngeal nerve, the salivary glands have plenty of neural tissues. Some researchers hypothesize that the tumor cells grow along the eural tissues. Some res” which serves as a conduit for their distant migration, whereas the other researchers believe that there are certain factor-based interactions between tumor cells and nerves, which provide a microenvironment suitable for tumor cell growth and proliferation around the nerves (15). In addition, many recent studies have suggested that cell signaling factors contribute to the interaction between tumor and nerve tissue; for example, by increasing the affinity of tumor cells for neural tissue (8). At present, the occurrence of neurotropic invasion is widely considered to involve a variety of microenvironmental regulatory factors, including chemokines and their receptors, brain-derived neurotrophic factor (BDNF) family proteins and their receptors, nerve growth factor (NGF) family proteins and their receptors, as well as matrix metalloproteinases (MMPs) and PNI-related cells (such as macrophages, astrocytes, and Schwann cells) (16).

In this review, we summarize the current knowledge concerning the characteristics of SACC in relation to the PNI, and then highlight the interplay between components of the tumor microenvironment and perineural niche, as well as their contributions to the PNI. Finally, we provide new insights into the possible mechanisms underlying the pathogenesis of PNI, with particular emphasis on the role of extracellular vesicles that may serve as an attractive candidate pathways in future studies.

## Anatomical Factors of SACC Perineural Invasion

PNI is a common clinical manifestation of various tumors, including pancreatic cancer, gastric cancer, prostate cancer, and head and neck cancer (17–20). PNI is distinct from PNS. Specifically, PNI is characterized at the microscopic level by the confined invasion of the tumor mass into nerves, while PNS refers to the clinico-radiological findings of distant distribution via the perineural space, or within the neural sheath and nerve itself (21). In 2009, Liebig et al. proposed that PNI of tumor cells can be defined as tumor cells near peripheral nerve fibers, surrounding them by at least 33%, or tumor cells infiltrating into any layers of nerves–endoneurium, perineurium, and epineurium (1). However, some scholars have divide neural invasion into epineural association, perineural invasion, and endoneural invasion based on the scope of neural invasion. Among them, endoneural invasion is an independent predictor of poor prognosis (22, 23).

The normal peripheral nerve consists of three layers of connective tissue with differing characteristics. The epineurium, the outermost fascial layer, is composed of the dense irregular connective tissues that binds individual nerve fascicles into a nerve trunk; the endoneurium is the innermost layer surrounding axons along with Schwann cells (SCs); the perineurium, the middle layer, is made of layers of flattened cells forming laminar structures wrapping around single nerve fascicles. Epineurium surrounds the entire nerve trunk, thus contributing to the tensile strength of the nerve, but it does not provide a barrier function. The existence of the tight junction (TJ) structures in perineurium and endoneurial vasculature forms the peripheral nerve barrier (24, 25). These structures have been recognized as shielding barriers to the paracellular diffusion of certain molecules and ions (26, 27). Recently, an increasing number of studies have indicated that TJ may act as a barrier against cancer invasion and metastasis (28–30). Therefore, we speculate that a possible explanation of PNI of malignant tumors may be that when tumor cells begin to invade peripheral never, the TJ structure of perineurium must first be disturbed and dismantled to facilitate penetration of the tumor cells.

## The Perineural Niche and Tumor Microenvironment in PNI

A study of neurological invasion, involving nerve cells, tumor cells, and stromal cells, investigated the neurotropic characteristics of prostate cancer and reported that these cell types could respond to secretory or intracellular cytokines, leading to neurologic attacks by tumors, in which axon growth is an important step (1). Axonal growth is complex and involves multiple factors, the most studied of which include chemokines and their receptors, the GDNF family and their receptors, and the NGF family and their receptors, along with matrix metalloproteinases (MMPs) and PNI-related cells (Table 1). Schwann cells are closely associated with the process of neural invasion, both prior to and during tumor invasion, and are therefore important for the initiation and development of PNI (16).

**Table 1:** Mediators in the perineural niche and tumor microenvironment in PNI.

**Factors**	**Interrelated factors**	**Role in PNI**	**References**
Perineurial cells	Tight junctions	The tight connections in the perineurial cells are the main players of the neural barrier function	(26, 27)
Other PNI-related cells	Schwann cells	Interact with preneoplastic cells	(31)
	TAMs	Positive correlation with PNI	(32, 33)
	Stellate cells	Induce the proliferation of cancer cell and associated with the generation of neuronal plasticity	(34, 35)
Nerve fibers	NGF secretion	Associated with NGF production and lymph node invasion in cancer	(36)
NGF family and receptors	NGF-TRKA	Positive correlations with PNI and poor prognosis in cancer	(37)
	BDNF-TRKB	Significantly correlated with advanced clinical stage, poor prognosis, PNI, vascular invasion, and distant metastasisin SACC; Have a role in the EMT process in SACC	(38, 39)
GDNF family and receptors	GDNF-GFRα1-RET	Induce tumor cell migration	(40)
Chemokines and receptors	CXCL12/CXCR4	Significantly correlated with PNI and increased secretion of MMPs	(41)
	CX3CL1/CX3CR1	Significant positive correlation with PNI and promote tumor migration and invasion	(42)
MMPs	MMP-2 and MMP-9	Degradation of ECM and basement membrane and involved in NGF–TRKA signaling and GDNF–RET pathway	(43)

### Perineurial Cells

A transmission electron microscopy study demonstrated that the thickness of the perineurium is ~10–25 microns, and that it consists of 8–15 concentric layers of flat perineurial cells (44). Each perineurial cell layer comprises flattened cells, linked by special connections that provide a barrier to diffusion. In addition, some intraneural blood vessels, with diameters of ~6–10 microns, were found close to the axons; these were composed of 6–8 layers of endothelial cells and formed a blood-neural barrier (BNB) with the perineurium (31). Previous studies have demonstrated that perineurial cells can control the integrity of the BNB by secreting various cytokines and growth factors, such as VEGF, BDNF, GDNF, bFGF, and Angiopoietin-1. Moreover, certain factors secreted by perineurial cells can also contribute to the regulation of the tight junction protein, claudin-5, in BNB endothelial cells, thus strengthening the barrier function of the BNB (32). These findings suggest that various factors in the neural microenvironment may regulate the BNB barrier function by affecting the tight junctions between perineurium cells.

Hence, it is important to fully understand the BNB that may represent the first line of defense against tumor PNI, in order to develop new options for the diagnosis and treatment of tumor PNI.

### Other PNI-Related Cells

Schwann cells (SCs) are the most common type of cells in peripheral nerves and, along with glial cells, play a key role in nerve repair and regeneration (33). Recent evidence suggests that the affinity of SCs for specific types of gastrointestinal cancer, as well as their migration in cancer, might precede any invasion of the neural environment, and thus play a role in PNI (34).

Tumor-associated macrophages (TAMs) interact with tumor cells and can modulate tumor growth, proliferation, metastasis, and prognosis via a series of cytokines, which are essential components of the tumor microenvironment (35). Endogenous macrophages also present molecules that participate in the balance and regeneration of peripheral nerves. Moreover, the production of GDNF by endothelial macrophages during PNI is promoted by CSF-1 secretion by pancreatic cancer (PC) cells. Notably, a clinical study has reported a correlation between macrophages and PNI (45).

Stellate cells are myofibroblasts, which can be activated by hypoxia, inflammation, and interactions with precancerous cells and cancer cells. Activated stellate cells promote PNI in pancreatic carcinoma (PC), and are a key contributor to connective tissue hypertrophy (36). The activation of stellate cells promotes the localized growth of PC in co-cultures of tumor cells and pancreatic astrocytes, as well as the propagation of PC cells in an *in-situ* model of PC (46).

### Nerve Fibers

Axons, also called nerve fibers, are thread-like projections that carry electrical signals between nerves and receptors in the skin, muscles, joints, and internal organs (47). Emerging evidence has shown that the invasion of tumor is dependent on the axon structure, and denervation can inhibit the growth and metastasis of tumor (48). Pundavela et al. have found that the presence of nerve fibers in breast cancers is relevant to lymph node invasion and the production of biologically active NGF (49) that can stimulate neuron outgrowth (axonogenesis or neo-neurogenesis), thus promoting PNI (50). Further, in order to investigate interactions between nerve fibers and tumor cells, Liu et al. established an *in vitro* model of PNI by co-culturing rat root ganglia (DRG) and human pancreatic cancer cell line (MIA PaCa-2). They found that these cancer cells could stimulate the outgrowth of neurites from DRG. Of note, while these neurites tended to migrate toward pancreatic cancer cell colonies, cancer cells also exhibited a trend of migrating along the contacting neurites simultaneously (37). This indicates that there may exist a reciprocal regulation loop between nerves and cancer cells. Moreover, it has been suggested that higher densities of nerve fibers within tumors were closely associated with poorer clinical outcomes in prostate cancer patients. However, in a mouse model of gastric cancer, the incidence and progression of tumors could be significantly reduced at denervation sites (either surgically or pharmacologically treated) of the stomach (51), again indicating a crucial role of nerves in the growth and progression of tumors (52). Cancer exosomes induced the innervation of the tumor and the exosomes from patients with head and neck cancer had neurite outgrowth activity, while the exosomes from the healthy control group did not (53). Nonetheless, due to the complexity of the tumor microenvironment, the role of nerve fibers in the tumor microenvironment is so far still an understudied research area that warrants further investigation.

### NGF Family and Receptors

The nerve growth factor (NGF) family mainly consists of neurotrophin 3 (NTF3), NGF, neurotrophin 4 (NTF4), and brain-derived neurotrophic factor (BDNF) (54). By binding to diverse receptors, NGFs can trigger multiple signaling pathways, which function in the regulation of cell growth, apoptosis, and neuronal formation. Besides, it not only has many important regulatory functions for the survival, growth and differentiation of nerve cells in the peripheral and central nervous system, but also plays a role in regulating the synthesis of neurotransmitters and neuropeptides in sympathetic and sensory nerve cells effect (38). Each NTF binds to tropomyosin-receptor kinases (TRK) receptor with high affinity, or to p75 neurotrophin receptor (p75NTR), with low affinity; NGF binds with high affinity to TRKA; BDNF and NTF4 both bind to TRKB receptors; and NTF-3 binds preferentially to TRKC (39).

Elevated NGF levels have been identified in PC, relative to adjacent normal tissues, with particular over-expression of TRKA in peripheral nerves. Additionally, there is a significant positive correlation between the expression of NGF and TRKA and the incidence of neural invasion, as well as a poor prognosis, in PC and cell co-culture models of PC (55).

BDNF is thought of as “brain fertilizer,” due to its potential roles in promoting the survival of existing neurons, as well as inducing the proliferation and differentiation of new neurons and synapses (56). However, BDNF and TRKB have been recently reported to be involved in the malignant progression of multiple cancers, including colon cancer, hepatocellular carcinoma, prostate cancer, and oral squamous cell carcinoma (40, 57). Jia et al. demonstrated that higher expression levels of BDNF and TRKB were significantly correlated with advanced clinical stage, poor prognosis, PNI, vascular invasion, and distant metastasis of SACC (41). Collectively, these findings implicate the involvement of BDNF/TRKB axis in PNI progression of SACC (58). Furthermore, the BDNF/TRKB axis is also reported to have a role in the epithelial-mesenchymal transition (EMT) process in SACC. And the study by Mei Zhang et al. proposed a correlation between PNI and MIF expression that MIF may promote the PNI of SACC by participating in cytoskeletal reorganization and pseudopod formation induced by Schwann-like cell differentiation of EMT and SACC cells (59). Despite the limited number of studies concerning the association between EMT and PNI, EMT is likely to play an invasive critical role in PNI progression, given the enhanced migration and abilities of cancer cells after EMT (42). Notably, there is a significant negative association between TRKB and E-cadherin expression in SACC specimens, supporting a potential role of the BDNF/TRKB axis in EMT during the development of PNI in SACC (58).

Recently, new evidence suggests that the PIK3K/Akt signaling pathway is associated with PNI in ACC. It has been found that NGF can activate PI3K/AKT pathway through phosphorylating AKT in SACC, thereby potentially stimulating scattering and migration of tumor cells which enhances the progression of PNI (60). Additionally, animal experiments have also shown that a high expression of Akt3 serves as a driving factor of salivary gland tumor progression (43). In addition, the NT-3/TrkC axis promotes the progression of PNI and the poor prognosis of SACC by regulating the interaction between SACC cells and SC. Interrupting the interaction between SACC cells and SC by blocking the NT-3/TrkC axis may be an effective strategy for anti-PNI therapy in SACC (61).

### GDNF Family and Receptors

The GDNF family, derived from the glial cell line, a group of neurotrophin polypeptides, which is comprises four members: neurturin (NRTN), persephin (PSPN), artemin (ARTN), and glial cell line-derived neurotrophic factor (GDNF). The GDNF family is secreted by neural tissues and binds with relevant receptors to trigger the differentiation of neuronal cells of the central and peripheral nervous systems. It works mainly in a paracrine manner and plays an important role in the development and maintenance of the central and peripheral nervous systems, kidney morphogenesis and sperm formation (42). GDNF, NRTN, ARTN, and PSPN bind to GFRA1 (GDNF family receptor alpha-1), GFRA2 (GDNF family receptor alpha-2), GFRA3 (GDNF family receptor alpha-3), and GFRA4 (GDNF family receptor alpha-4), respectively (42). It has been suggested that PNI may be mediated through the secretion of GDNF by nerves and further activation of tumor cell surface Ret proto-oncogene (RET) receptors. GFRA1 functions as a co-receptor with RET, both of which are required for GDNF interaction. He et al. have found that DRG neurons can release soluble GFRA1 that may enhance RET phosphorylation and tumor cell migration toward GDNF, even when tumor cell expression of GFRA1 is absent (62). Moreover, in another previous study, a high level of GDNF expression was detected in SACC cells and adjacent nerve fibers, which was demonstrated to be associated with an increase in matrix-degradation during PNI progression (63).

### Chemokines and Receptors

Chemokines and their receptors have critical roles in the growth and invasion of tumor cells. CXCL12/CXCR4 (C-X-C Motif Chemokine Ligand 12/C-X-C Motif chemokine receptor 4) signal transduction is a candidate for participation in inter-tumor interstitial interaction and has various functions, such as the regulation of cell proliferation, invasion, EMT, metastasis, and angiogenesis in multiple types of malignancy (64). In PC cells, MMP-9 secretion in response to CXCR4 stimulation may contribute to the process of PNI, via enhancement of extracellular matrix (ECM) degradation (65). Furthermore, CXCL12 significantly increases the NGF expression in PC cells and promotes neuronal regeneration by binding to the CXCR4 receptor. An *in vitro* model showed that the elimination of the CXCL12/CXCR4 signaling pathway led to the suppression of chemotactic migration between PC cells and nerve cells; by blocking the CXCL12/CXCR4 pathway, PC cell neurotropism decreased significantly (66). Therefore, CXCL12/CXCR4 signaling is significantly correlated with PNI. Moreover, Thomas et al. performed CXCR4 immunohistochemical staining and semiquantitative scoring on the tumor tissue of 73 head and neck adenoid cystic carcinoma (AdCC) patients and found that high CXCR4 expression in AdCC is associated with an increased risk of local recurrence (67).

CX3CL1 (C-X3-C Motif Chemokine Ligand 1) is found to be abundantly produced and released by neurons. An elevated expression level of its receptor, CX3CR1 (C-X3-C Motif Chemokine receptor 1), has been implicated in the development of PNI and earlier recurrence of numerous cancers, such as PC, gastric cancer, and prostate cancer (68–70). Equally important, both *in vitro* co-culture and *in vivo* PNI models demonstrated that nerve cells, including Schwann cells and neurons, express CCL2 (C-C motif chemokine ligand 2) which capable of inducinge the migration of CCR2 (C-C Motif Chemokine receptor 2) expressing cancer cells toward these nerves, ultimately promoting PNI progression (16, 71, 72). Role of CX3CR1/CX3CL1 axis in primary and secondary involvement of the nervous system by cancer.

### Matrix Metalloproteinases (MMPs)

MMPs are a family of endopeptidases responsible for the degradation of the ECM and tissue remodeling. The expression of certain types of collagen has been confirmed in peripheral nerves, including collagen IV, the main component of the basement membrane of Schwann cells (73). MMP2, MMP7, MMP9, and all type IV collagenases secreted in response to NGF or GDNF, are likely to be involved in PNI (74). In addition, the overexpression of MMP2 by myofibroblasts has been documented in SACC, exhibiting high-grade PNI.

These results indicate that nerves can indeed provide an appropriate environment for tumor growth and the reciprocal interaction positively influence the growth of both nerves and tumors. Although the accuracy of a “path of low resistance” theory is still up for debate today, emerging evidence indicates that the PNI phenomenon is more like a process of active invasion rather than simple diffusion.

## PNI Models

As mentioned above, PNI is very closely correlated with the prognosis of patients with SACC. Although it was discovered over a century ago, we still know little about the molecular mechanisms involved in the PNI process. *In vitro* models of these complicated disease processes are very difficult to create. To gain a comprehensive understanding of the contribution of a series of soluble factors to PNI, some researchers have attempted to create *in vitro* models, using highly controlled experimental settings. For example, Ayala et al. established an *in vitro* model, where murine DRGs were co-cultured with prostate cancer cells in Matrigel (75). Once suspended in Matrigel, the axons from the DRGs could spread in all directions. These axons grew toward tumor colonies and were gradually invaded by cancer, which mimics the clinical observation of the typical centripetalism of PNI spread. Using this model, Ayala et al. first described the symbiosis exhibited between cancers and nerves in PNI, in which both gain a growth advantage when co-cultured (76). Although the use of this model can better mimic the invasion of tumor cells into nearby nerves, it is not able to replicate the highly complex microenvironment of *in vivo* perineural niches. Of note, Deborde et al. used Live-imaging technology to investigate the interactions between cancer cells and Schwann cells *in vitro* coculture and *in vivo* murine models of PNI. They found that neither soluble factors secreted by Schwann cells, nor empty tunnels established by Schwann cells were capable of mediating cancer cell invasion, whereas physical contact between Schwann living cells and cancer cells facilitated invasion process. This model provides a new insight into the analysis of the complex *in vivo* characteristics of Schwann cells in enhancing PNI (77). Currently, a murine sciatic nerve model is widely used to explore PNI; tumor cells are injected into the distal sciatic nerve of mice to establish an *in vivo* PNI model. Under gross observation, the sciatic nerve is seen enlarged; histologically, tumor infiltration of the sciatic nerve can be visualized by hematoxylin and eosin staining and immunohistochemistry of pathological tissue sections. Notably, the effects of PNI can be analyzed by monitoring hind limb motor performance, such as the hind paw width of the mice, as well as sciatic neurological score. More importantly, this model, with the genetic manipulation of mice and/or use of different types of cancer cells, can be applied to study cellular and molecular mechanisms involved in PNI and the effects of therapeutic agents on neural invasion (78). These emerging models of PNI strongly suggested there is an extensive signaling interaction between the nerves and invading tumor cells. Recently, an *in vivo* PNI model has been developed to unravel molecular mechanisms of nerve–tumor interactions. Briefly, rat DRGs are transplanted onto the chick embryo chorioallantoic membrane, followed by the transplantation of human head and neck squamous cell carcinoma (HNSCC) cells adjacent to the DRG. This system can replicate a pro-angiogenic tumor microenvironment observed in carcinogenesis, where the newly formed vasculature provides nourishment for both DRGs and tumor cell grafts (79).

In the past few decades, three-dimensional (3D) printing technology has received widespread attention. Now 3D scaffolds fabricated by 3D bioprinting of biomaterials (bioinks) can be used for the regeneration and reconstruction of complex tissues and organs. Likewise, printed 3D tumor models have also been developed to simulate the *in vivo* tumor microenvironment (80). For instance, using 3D projection printing, an *in vitro* 3D micro-chip in a hydrogel was built, with the objective of simulating 3D vascular morphology of *in vivo* microenvironment, by which the behavior of both cancer and non-cancer cells can be monitored and analyzed (81). In addition, Valentina et al. reported that 3D bioprinting, combined with induced pluripotent stem cells (iPSCs) could effectively control the spatial distribution of cells (e.g., neurons); thus creating a more reliable *in vitro* model (82). Although the applications of this technology in the construction of *in-vitro* 3D tumor and neural tissue models are, so far, in the research stage, they provide some new insights into the study of the underlying mechanisms of PNI pathogenesis and progression.

## Possible Mechanism of PNI and Conclusion

**Figure 1:**
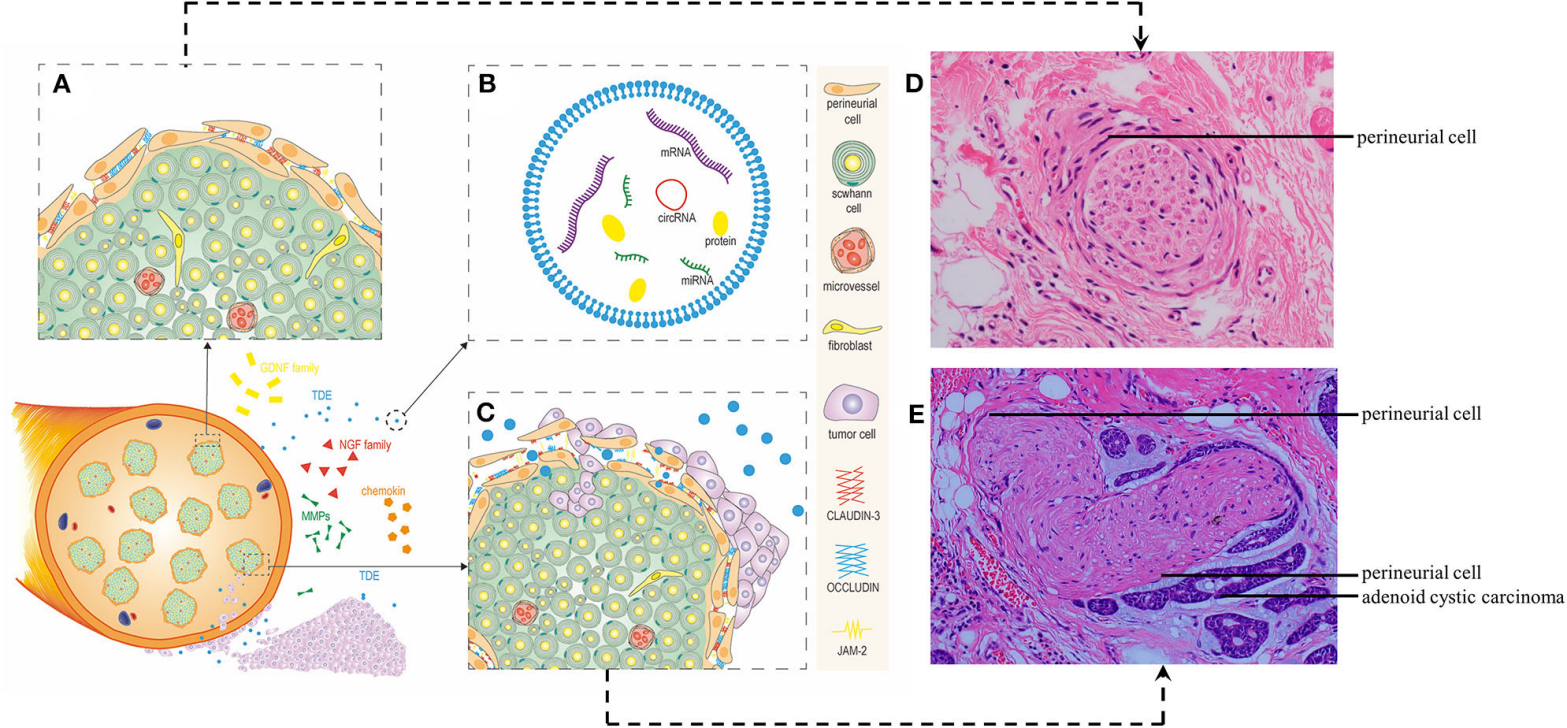
The perineural niche and tumor microenvironment in PNI. **(A)** Diagram showing the architecture and main cellular components of a normal axon. The perineurium that composed of several layers of perineurial cells protects the axon. The perineurium that surrounds individual Schwann cell, micro-vessel, fibroblast. **(B)** Diagram showing the brief structure of tumor derived-exosomes(TDE). TDE contains mRNA, miRNA, protein and other substances, which can be transferred to their target cells, directly or indirectly changing the biological function of recipient cells. **(C)** Diagram showing the architecture and main cellular components of an axon while tumor invades the nerve. After the effect of multiple factors, such as chemokines, GDNF family, NGF family, MMPs and PNI-related cells, the tight connections between the perineurial cells the and the tumor invades the nerve. **(D)** Diagram show the corresponding H&E-stained image of normal nerve. **(E)** Diagram show the corresponding H&E-stained image of tumor infiltrating nerve by the adenoid cystic carcinoma.

Due to its insidious onset, its susceptibility to PNI, and high recurrence rates, SACCs are common malignant neoplasms of head and neck tumors. Furthermore, PNI is closely related to patient prognosis. Although many studies have investigated the mechanisms underlying PNI, the precise details remain unclear. In recent years, researchers have begun to investigate the concept that nerves can reciprocally facilitate cancer invasion and progression (83), representing a paradigm shift in our understanding of the mechanism of PNI. Based on current research, we consider that although anatomical factors, cells, nerve growth factors (NGF, BDNF, etc.), chemokines in the tumor microenvironment are involved in the development of PNI, there are other factors that may also contribute to PNI, such as extracellular vesicles (EVs) derived from tumor cells, or other cells in tumor microenvironment and perineural niches (Figure 1).

Exosomes, known as the smallest subgroup of EVs, are secreted by cells when multivesicular bodies (MVBs) fuse with the plasma membrane (84). Many previous studies have shown that not only tumor cells produce more exosomes than normal cells, but also tumor-derived exosomes (TDE) containing a diverse set of proteins, mRNA, miRNA, and lipids, can “educate” interstitial cells, endothelial cells, inflammatory cells, and immune cells, in order to regulate the tumor microenvironment and promote tumor growth, invasion, metastasis and angiogenesis (53, 85). Melo et al. (86) found that breast cancer cells can produce mature exosomal miRNAs via a non-cell-dependent pathway, which regulates the phenotype of normal epithelial cells and accelerate their proliferation, and can also be used to generate tumors in nude mice. The development of distant metastases requires primary tumors to break through the basement membrane and penetrate the lymphatic or vascular circulation. Notably, invasive precancerous epithelial cells can overcome this physical constraint by obtaining invasive and migration properties through EMT. Taverna et al. (87) found that exosomal miR-126 derived from chronic myeloid leukemia cells can alter the adhesion and migration ability of recipient cells. Moreover, other studies have confirmed that exosomal miR-105 is specifically expressed and secreted by metastatic breast cancer cells via exosome secretion, and can be transferred to endothelial cells, thereby facilitating cancer metastasis by disrupting the vascular endothelial barrier (88). Hoshino et al. found that tumor-derived exosomes could direct organ-specific metastasis via exosomal integrins (ITGs) (89), based on their intrinsic organotropic homing ability and capacity of initiating pre-metastatic niche formation at foreign sites. Moreover, not only can tumor cells alter the cellular physiology of both surrounding and distant microenvironment through TDE, but other cells in tumor/pre-metastatic microenvironment can also target tumor cells via exosomes, leading to the co-evolution of tumor cells and their microenvironment during tumor dissemination and metastatic outgrowth. A recent study demonstrated that exosomes released by astrocytes could induce an intercellular transfer of PTEN-targeting microRNAs to metastatic tumor cells (90). This finding indicates that exosomes from non-neoplastic cells may also promote the adaptation of disseminated tumor cells to target organs, suggesting the involvement of dynamic bidirectional crosstalk in the pre-metastatic microenvironment. Many previous studies have shown that EVs participate in the dissemination of numerous primary cancer cells, including breast cancer, gastric cancer, colon cancer, liver cancer, malignant melanoma, etc. (91). For instance, one recently published study reported that head and neck tumor-derived exosomes could induce tumor innervation that was enhanced by the exosome-packaged axonal guidance molecule, EphrinB1. This indicates that interventions targeting exosome biogenesis and release may be of therapeutic value against PNI (92). Of particular note, in our previous study, we found that the exosomes derived from the adenoid cystic carcinoma cell line (SACC-83) could be uptaken by human umbilical vein endothelial cells (HUVECs), and destroyed the vascular endothelial barrier (93). Considering that primary tumor-derived EVs can destroy epithelial cell tight junction assembly to induce EMT, and trigger vascular permeability to allow cancer cell dissemination, and “educate” pre-metastatic sites in distant organs, we propose that EVs may also contribute to the disruption of the perineurium barrier and education of the perineural sites into a tumor-promoting microenvironment. It may provide new directions and ideas for further study of PNI pathogenesis in the future. Further, although some research groups have described the involvement of tumor and stroma-derived EVs in the different stages of the metastatic cascade, there is very little information concerning the regulation of tumor cell invasion and metastasis by extracellular vesicles in SACC as well as in PNI.

Of particular note, Galanin is a classic neuropeptide that can function in a variety of physiological processes, such as food intake, nociception, and blood pressure regulation, and it can also act as a growth factor for neurons. Galanin treatment has a tumor-reducing effect in a mouse model of gastrointestinal cancer, and in animal experiments with adenoma formation, galanin appears to be a growth factor that promotes both proliferation and tumor formation (94). In addition, Laminin-5 and insulin-like growth factor-II mRNA binding protein-3 (IMP3) have a good prognostic correlation in various malignancies. Studies have shown that they are also positively expressed in preoperative biopsy specimens of OSCC patients with PNI, implying that preoperative assessment of factors associated with PNI may assist the clinician in the selection of the optimal treatment strategy (95). Nonetheless, whether they can be used as molecular markers of PNI in SACC still needs to be further evaluated.

It's worth mentioning that PNI has been rarely studied in patients with SACC. Emerging evidence for its role as a prognostic factor in SACC is conflicting. Due to the indolent clinical course of SACC, the evaluation of a patient with this malignant tumor is hampered. Furthermore, most previous studies were limited by their small sample size, relatively short follow-up period, mixed pathologies and variable histological sampling of salivary glands, and inadequate reporting of PNI as well (96). Genomic Alterations may also be one of the influencing factors of PNI in SACC. The study found that peripheral nerve invasion was found to be fused with MYB gene with or without copy number changes. MYB-NFIB gene fusion and related GA are associated with ACC peripheral nerve involvement (97). Nonetheless, several studies have found that specific PNI features are indeed closely related to SACC metastasis, locoregional recurrence, long-term survival, and a patient's quality of life (98).

Accordingly, we consider that there are two critical steps in developing strategies for prevention and early therapeutic intervention of PNI: (1) identifying the molecular targets in the microenvironment of perineural niches which are necessary for PNI development, and (2) finding specific agents against these molecular targets. Therefore, a deeper investigation of underlying mechanisms of PNI should not only clarify the occurrence, proliferation, invasion, recurrence, and metastasis of SACC, but also provide potential targets for the diagnosis and therapy of neurological disorders and diseases.

In conclusion, multiple processes may result in damage at cancer-neural interfaces. There is reciprocity in these interactions, which confers growth and migration advantages to cancer. An in-depth understanding of the molecular mechanism of PNI is essential for developing therapeutic strategies that not only target cancer cells, but also target neural microenvironments as well.

## Author Contributions

XYi and JH conceived the idea of writing the review. XL wrote the review. XYa assisted to edit. CZ and JZ prepared the Tables and Figures. All authors contributed to the article and approved the submitted version.

## Conflict of Interest

The authors declare that the research was conducted in the absence of any commercial or financial relationships that could be construed as a potential conflict of interest.
